# Fabrication of SA/Gel/C scaffold with 3D bioprinting to generate micro-nano porosity structure for skin wound healing: a detailed animal in vivo study

**DOI:** 10.1186/s13619-022-00113-y

**Published:** 2022-05-01

**Authors:** Changmei Niu, Liyang Wang, Dongdong Ji, Mingjun Ren, Dongxu Ke, Qiang Fu, Kaile Zhang, Xi Yang

**Affiliations:** 1 Novaprint Therapeutics Suzhou Co., Ltd, Suzhou, 215000 China; 2 Shanghai University of Engineering Science, Shanghai, 201620 China; 3 Department of Burns and Plastic Surgery Affiliated Suzhou Hospital Of Nanjing Medical University, Suzhou, 215000 China; 4 State Key Laboratory of Mechanical System and Vibration, School of Mechanical Engineering, Shanghai Jiaotong University, Shanghai, 200240 China; 5 The Department of Urology, Affiliated Sixth People’s Hospital, Shanghai JiaoTong University, Shanghai, 200235 China; 6 Shanghai Eastern Institute of Urologic Reconstruction, Shanghai, 200000 China

**Keywords:** 3D bioprinting, Skin tissue engineering, Skin scaffold, Hydrogel

## Abstract

Bioprinting has exhibited remarkable promises for the fabrication of functional skin substitutes. However, there are some significant challenges for the treatment of full-thickness skin defects in clinical practice. It is necessary to determine bioinks with suitable mechanical properties and desirable biocompatibilities. Additionally, the key for printing skin is to design the skin structure optimally, enabling the function of the skin. In this study, the full-thickness skin scaffolds were prepared with a gradient pore structure constructing the dense layer, epidermis, and dermis by different ratios of bioinks. We hypothesized that the dense layer protects the wound surface and maintains a moist environment on the wound surface. By developing a suitable hydrogel bioink formulation (sodium alginate/gelatin/collagen), to simulate the physiological structure of the skin via 3D printing, the proportion of hydrogels was optimized corresponding to each layer. These results reveal that the scaffold has interconnected macroscopic channels, and sodium alginate/gelatin/collagen scaffolds accelerated wound healing, reduced skin wound contraction, and re-epithelialization in vivo. It is expected to provide a rapid and economical production method of skin scaffolds for future clinical applications.

## Background

Skin injuries are caused by many reasons, such as burns, surgical infections, cancer chemotherapy, diabetic feet, and chronic ulcers. With the increase in morbidity and mortality, the quality of life of patients was seriously affected (Cubo et al., 2016, Chouhan and Mandal, 2020, Huang et al., 2016). Although the skin has the ability to regenerate, the previous function of the skin was hardly restored perfectly. Besides, the self-healing ability of the wound is weak and has permanent scars (Low et al., 2019). To improve the efficiency of wound healing, some methods and skin substitutes have been investigated, such as allograft, autograft, xenograft, and tissue-engineered skin products (Yang et al., 2019). The well-known method is to obtain autologous grafts from other parts of the patient’s body in the clinic. Although this method reduces immune rejection during transplantation, the infection and damage to other soft tissues may increase (Sheikholeslam et al., 2018, Climov et al., 2016). Therefore, transplantation and tissue repair are immensely compromised using allogeneic organs (Matai et al., 2020, Zhou et al., 2020). In addition, the concept of skin substitutes is proposed, which is a group of heterogeneous substances that help the wound to close temporarily or permanently based on the extent of wound coverage (Shores et al., 2007).

In the past few decades, the field of tissue engineering and regenerative medicine has been developing rapidly (Beheshtizadeh et al., 2019, Mason and Dunnill, 2008). However, there are some limitations for traditional tissue engineering techniques in general clinical treatment. For example, the cells seeded on the scaffold are not attached uniformly and firmly, causing the death of cells. Currently, the ideal skin substitutes have not been found on the market (Shores et al., 2007). 3D bioprinting, as state-of-the-art manufacturing technology, is leading a revolutionary change in global medicine and has recently achieved gratifying results in clinical translation. (Urciuolo et al., 2020, Shi et al., 2018, Albanna et al., 2019). The application of bioprinted skin scaffold includes wound healing, model, cosmetic testing, and drug loading testing (Admane et al., 2019, Yan et al., 2018). In 2008, a multilayer scaffold loaded with human skin fibroblasts and keratinocytes was fabricated by the three-dimensional freeform fabrication (FF) technique. Although the dermal/epidermal-like layers were formed, the inhomogeneous distribution in depth was presented (Lee et al., 2009). Due to the rapid and effective creation of the hierarchical relationship of biological structures, 3D bioprinting has recently attracted increasing interest from researchers around the world (Ozbolat, 2015). The high resolution of 3D bioprinting technology, biomaterials, and multifunctional cells reproduces natural extracellular matrix components and simulates the biological functions of natural tissues (Murphy and Atala, 2014, Kim et al., 2017). Moreover, there are many advantages with simplicity, rapid manufacturing, and precise cell deposition positioning for this technique, compared with other tissue engineering manufacturing technologies (Yuan et al., 2016).

Bioinks, as the key for 3D bioprinting, is usually defined as a formula containing biological materials and biologically active ingredients. Also, bioinks are suited for automated manufacturing technology (Groll et al., 2018). There are many types of bioinks, such as hydrogels, acellular matrix, cell aggregates, high molecular polymers. Most importantly, based on naturally-derived polymers, bioinks include sodium alginate, gelatin, chitosan, silk fibroin, collagen, fibrin, hyaluronic acid, which have high similarities with the natural extracellular matrix (Vijayavenkataraman et al., 2016). Additionally, the basic parameters of the whole printing process include printing methods, biomaterials, and cells (Ouyang et al., 2016). Herein, our primary purpose is to optimize the ratio of biomaterials and the construction of scaffolds. The combinations of gelatin/sodium alginate hydrogels are more commonly used in extrusion bioprinting (Ouyang et al., 2016, Yao et al., 2019, Liu et al., 2019, Sarker et al., 2014). Ouyang et al., (2016) tested the rheological properties of gelatin/sodium alginate hydrogels. They found that the printing performance of gelatin/sodium alginate has deteriorated with the extension of gel time. Meanwhile, with the concentration of bioinks increasing, the entanglement of polymer chains inhibits cell migration and proliferation, resulting in a decrease in cell viability. Therefore, this is reasonably instructive for printing with cells and adjusting the printing process parameters.

In this study, sodium alginate/gelatin/collagen (SA/Gel/C) hydrogel as the bioinks constructed a bionic skin model in vitro, based on the actual situation of the skin. We are committed to exploring a stable and bionic skin scaffold through optimizing the concentrations and printing performance of bioinks. In this work, the hydrogel concentrations are optimized to simulate the structure of the skin. And the printing parameters are also adjusted. The preparation of the full-thickness skin scaffold was manufactured by extrusion-based 3D bioprinting. Furthermore, the full-thickness skin scaffold was applied to the skin defect model of pigs. The repair effect was evaluated by histological analysis and immunofluorescence staining.

## Results

### Preparation of the printable skin bioinks

**Fig. 1:**
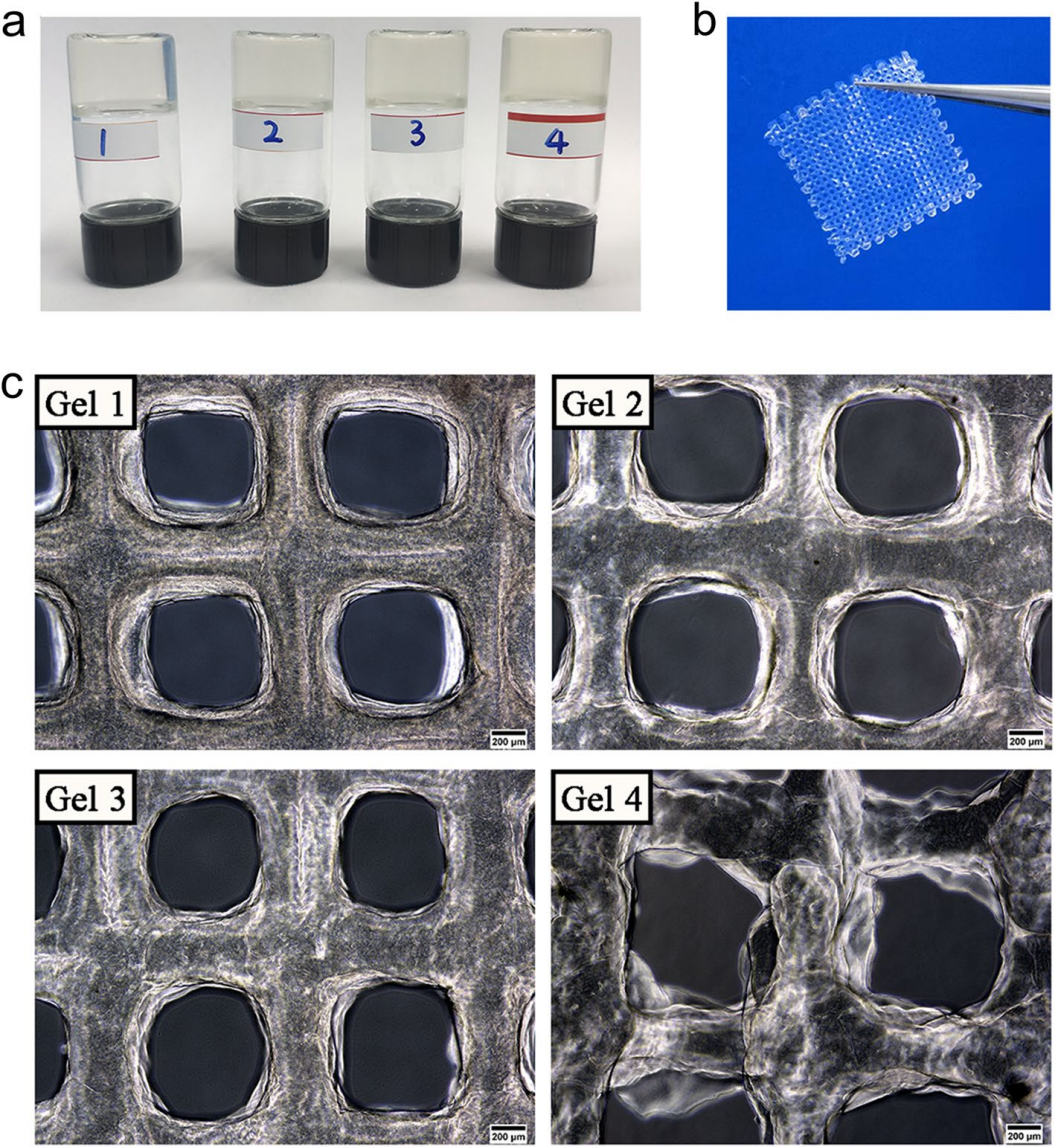
Printing test of hydrogel bioink. **A** The preparation of four different bioinks (Gel 1, Gel 2, Gel 3, and Gel 4, respectively). Gelatinization was occurred through the physical crosslinking at laboratory temperature. **B** The multilayer skin scaffold for printing. **C** The lines of the scaffold were observed under an optical microscope

The evaluation standard of printable hydrogel bioink is to form a continuous and uniform cylindrical line during the extrusion process. In addition, the hydrogel bioink cannot be in a droplet or completely gel state (Rastin et al., 2020). To prevent the SA/Gel/C scaffold from collapsing the hydrogel solution has been placed for several hours at the laboratory temperature (19 °C) upside down (Fig. 1a). Moreover, the hydrogel in this reversibly crosslinked state was fragile and unstable, and the flowing liquid was initialized again at 37 °C. Therefore, the temperature of the printing platform was between 18 and 22 °C. Meanwhile, the stable structure of the scaffold was maintained by the chemical crosslinking of SA and Ca^2+^. The prepared SA/Gel/C hydrogel was extruded at a constant volume to evaluate the formation of lines. Figure 1b shows the printed grid structures. The grid shape was observed and measured linewidth under an inverted microscope. The grid shapes of four hydrogels are shown in Fig. 1c. The results showed that the line of the grid was extruded unevenly and accumulated on the corner. The viscosity of Gel 4 (4% gelatin, 1% SA and 1% C) was extremely high during extrusion printing because of the concentration of gelatin and the affection of temperature. With the concentration of gelatin concentration decreasing, the line shapes were extruded with smooth and uniform. Therefore, Gel 1(dense layer; 1.8% gelatin, 2.5% SA and 0.5% C), Gel 2 (epidermis layer, 2% gelatin, 2% SA and 1% C), Gel 3 (dermal layer, 2.3% gelatin, 1.5% SA and 0.5% C) bioinks were chosen for skin printing to obtain a structure with better shape integrity.

### Design of the bioprinting skin scaffold and structure characterization

**Fig. 2:**
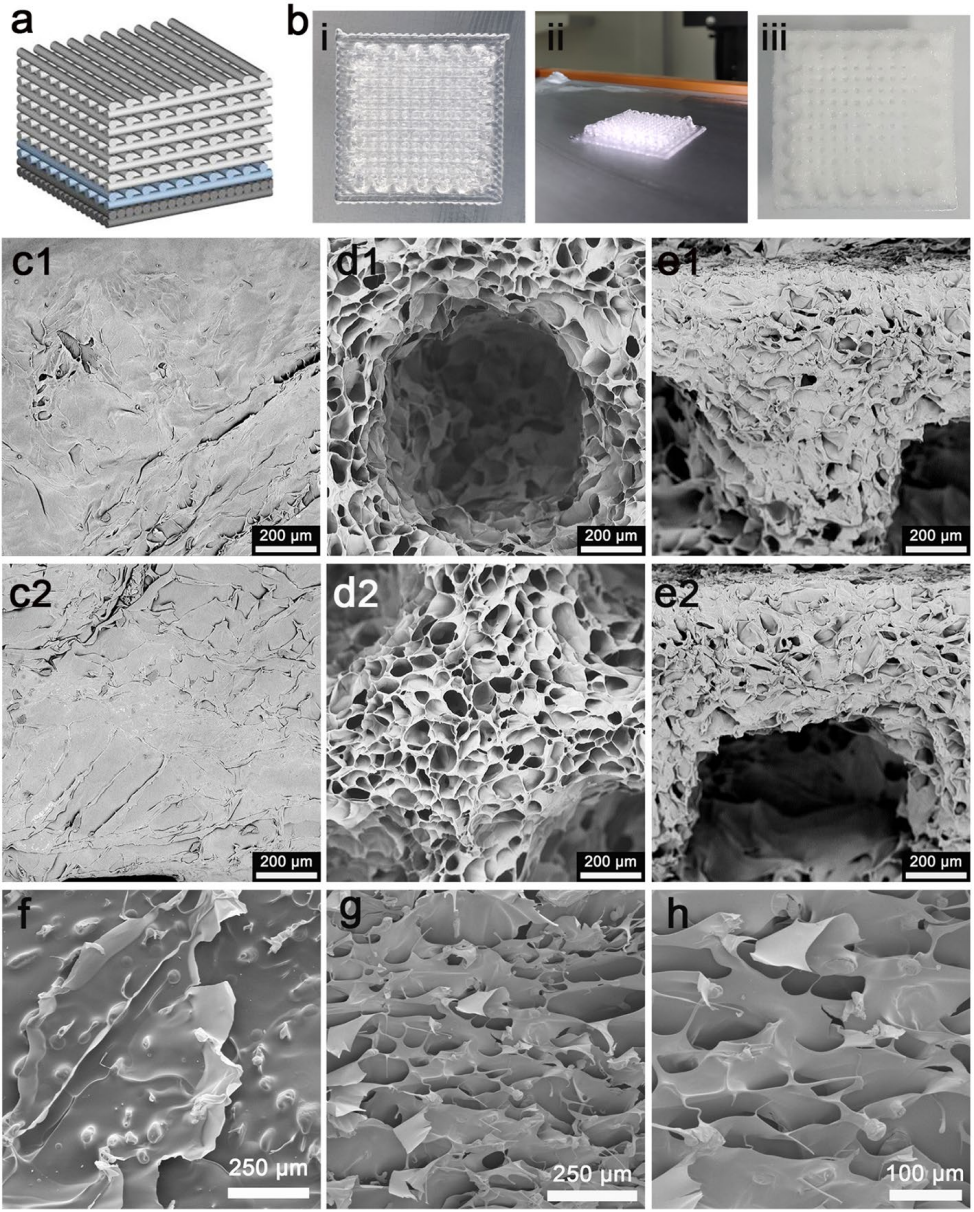
Structure of the 3D bioprinted SA/Gel/C scaffold. **A** A schematic diagram of the structure of the bionic skin scaffold. **B** The appearance of the scaffold: (i) the top view and (ii) the side view of the printed scaffold, and (iii) the freeze-dried scaffold includes the dermis layer. Scanning electron microscopy images of the scaffold without fibroblasts. From left to right, the upper layer (**C**1, **C**2), lower layer (**D**1, **D**2), and side view (**E**1, **E**2). SEM images of fibroblasts on scaffolds. On day 1 after culture, fibroblasts on the upper layer (**F**) and sectional view (**G** and **H**)

The full-thickness skin scaffolds were prepared with a gradient pore structure constructing the dense layer, epidermis layer, and dermis layer by different ratios of bioinks (Fig. 2a). As shown in Fig. 2b, (i) the top view and (ii) the side view of the printed scaffold, and (iii) the distribution and arrangement of porous structures were uniformly and neat after freeze-drying. The scanning electron microscopic image of the freeze-dried scaffold showed that the surface of the dense layer was compact and not perforations, which protected the wound surface and maintained a moist environment (Fig. 2c). The lower layer and sectional view were shown in Fig. 2d and e, respectively. The macroscopic pores structure of the epidermis and dermis is conducive to the adhesion and proliferation of cells, the transmission of nutrient substances, and the excretion of metabolic wastes. The SEM results of the scaffold showed that the pore size was about 60.52 ± 4.73 μm. The surface of the scaffold was dense, with a large number of adhered fibroblasts, which were indicated by the red arrow and attached to the scaffold (Fig. 2f). However, there were not too many cells adhering to the surface of the pores (Fig. 2g and h).

### Cell viability assay

**Fig. 3:**
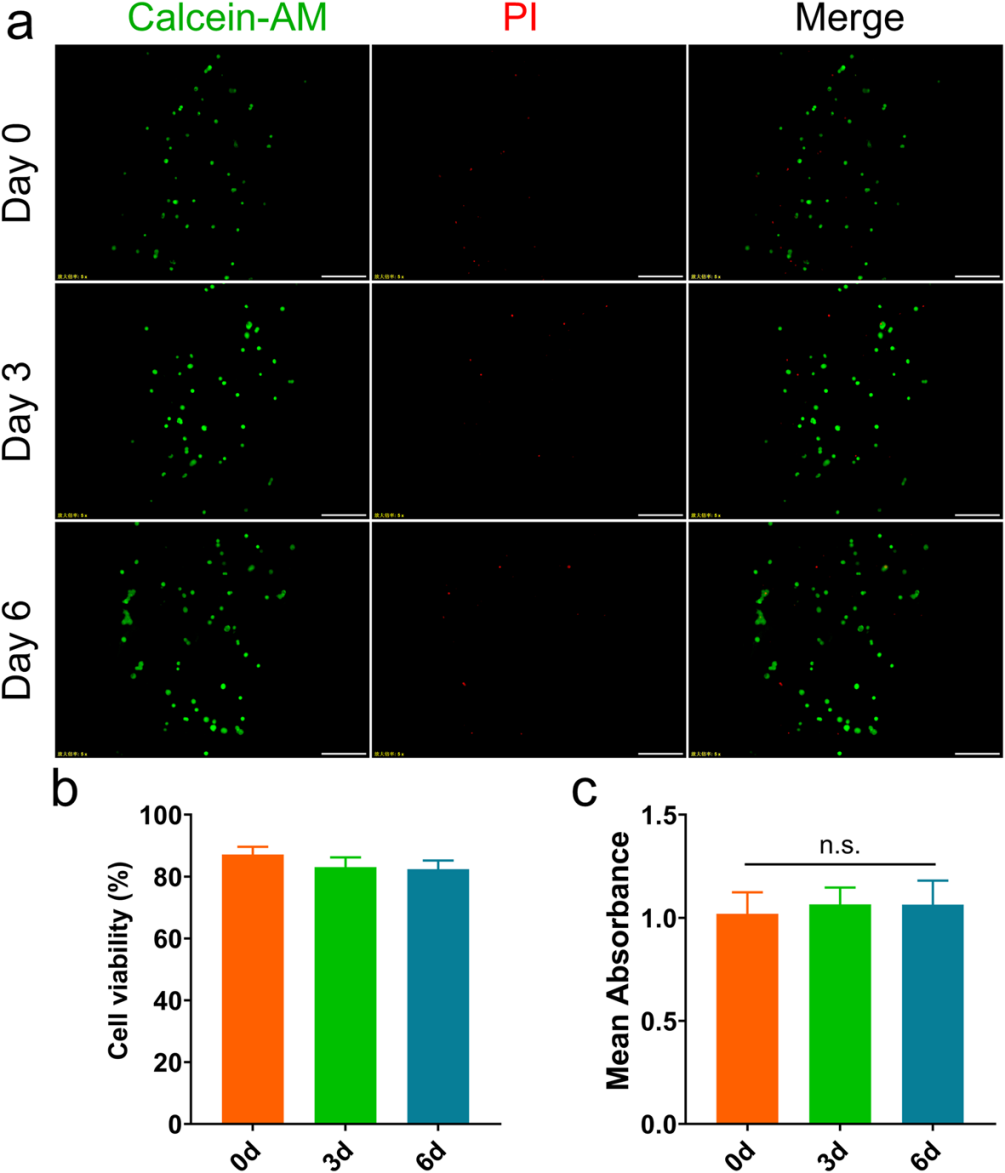
Live/Dead staining at 0, 3, and 6 days after fibroblasts encapsulation in the printed scaffold. **A** Images of the printed fibroblasts-laden SA/Gel/C hydrogel after days 0, 3, and 6 culturing. Living cells are stained green and dead cells red. Scale bar 500 μm. **B** Cell viability of fibroblasts in the printed SA/Gel/C hydrogel at days 0, 3, and 6. **C** MTT assay of fibroblasts on scaffolds at days 0, 3, and 6

The non-cytotoxicity of materials is essential for further clinical applications. The live/dead images of encapsulated fibroblasts at days 0, 3, and 6 were shown in Fig. 3a, with the live cells being green fluorescent and the dead cells being red fluorescent. In the case of the printed and crosslinked hydrogels, the live/dead staining showed that the cell viability of fibroblasts was up to 90% at day 0. However, the cell viability of fibroblasts decreased slightly on days 3 and 6. Moreover, the cells encapsulated in hydrogel were difficult to recover within the culture period. Interestingly, the cells had formed clusters on day 3. Although cell viabilities (> 80%) were maintained at an equally relatively good level until the end of the culture, the overall trend of cell viability was a slight decrease (Fig. 3b). As shown in Fig. 3c, the relative absorption between hydrogel scaffold and control group was not statistically significant. The result demonstrated that the SA/Gel/C scaffold was nontoxic to the fibroblasts.

### Skin scaffold for full-thickness skin defect repair in a porcine model

**Fig. 4:**
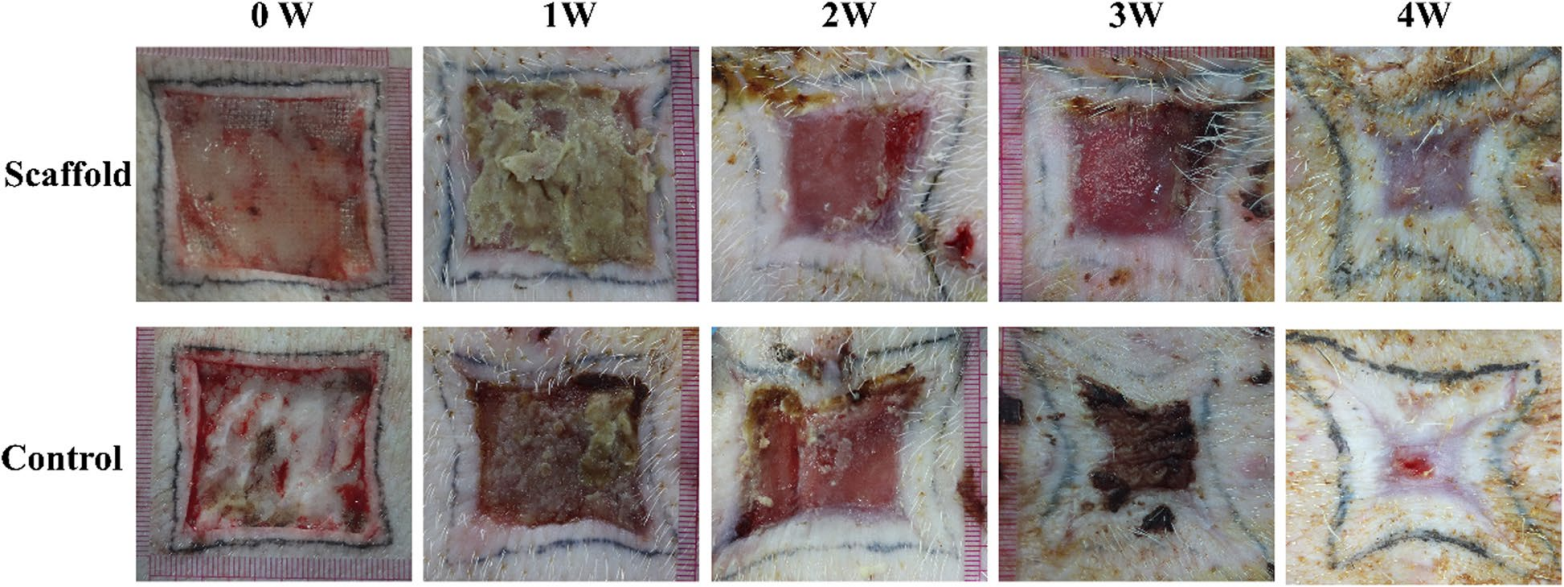
The gross morphology of the wound healing process for 4 weeks. Full-thickness skin defects were divided into two groups: the scaffold group and the control group, respectively

To further verify the safety and function of the scaffold, the large animal model was used to simulate the experiment clinically. The porcine full-thickness skin defect model with a wound area of 4 cm × 4 cm was prepared, as shown in Fig. 4. The gross morphology of the scaffold group showed that the scaffold materials adhered to the wound surface, and the bottom granulation tissue was grown. Then, the degradation of scaffold had occurred, compared with the control group at one week postoperatively. There was no adhesion between the wound and the gauze dressing. Also, there was no inflammatory exudate on the wound surface. Most importantly, the wound contracture of the scaffold group was significantly smaller than that in the control group at 4 weeks postoperatively. The scaffold on the wound has basically degraded. The wound depth tended to be flat and gradually returned to normal skin. The results showed that the wound area of the experimental group was significantly reduced at 2 weeks postoperatively. The wound had healed completely in the SA/Gel/C scaffold group for 4 weeks postoperatively, compared with the control group. The area around the wound without the SA/Gel/C scaffold has healed, and the middle wound has healed yet. This study demonstrated that the rapid closure of wounds could be realized by using the SA/Gel/C scaffolds.

### Histological evaluation

**Fig. 5:**
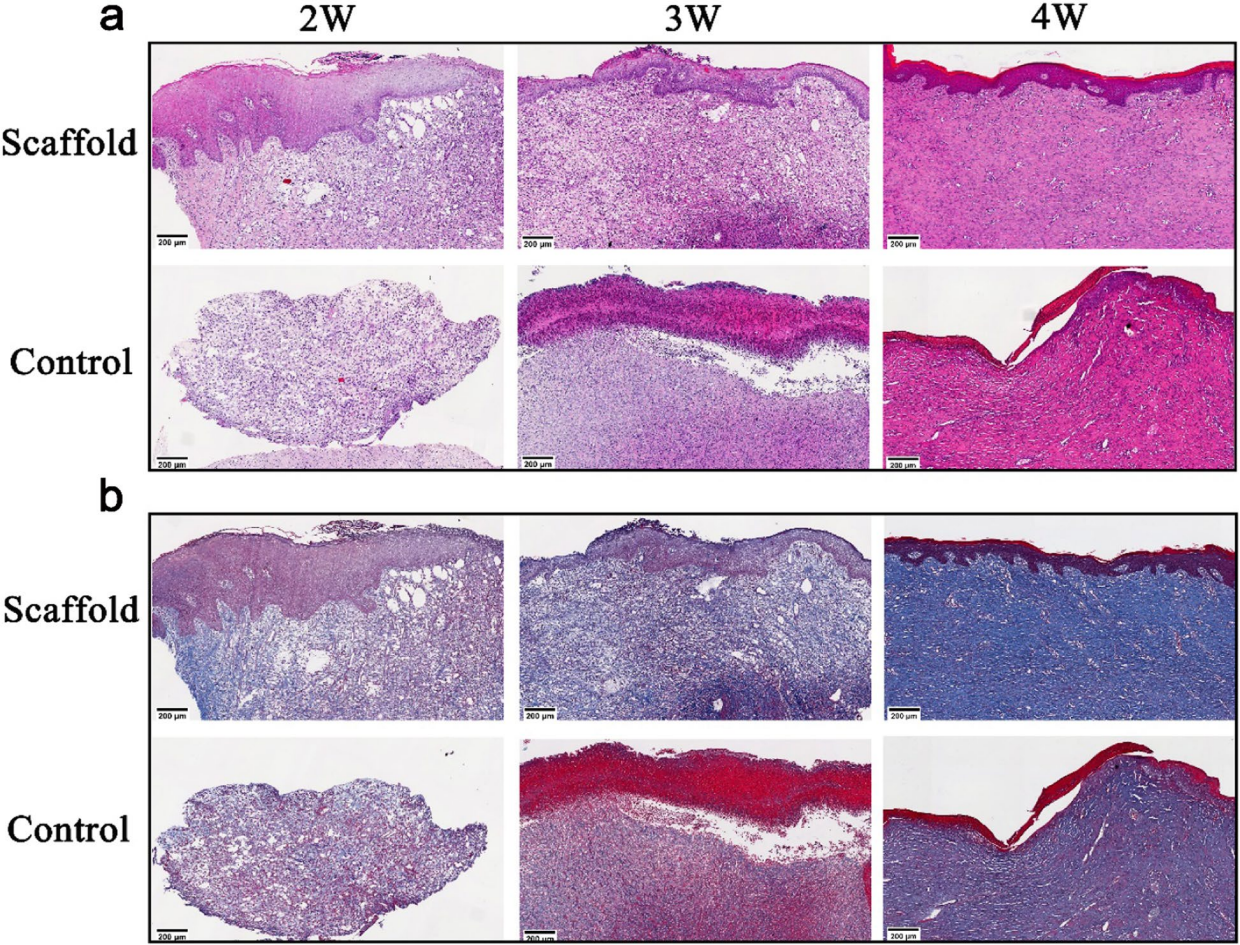
Representative images of H&E staining (**A**) and Masson’s trichrome staining (**B**) of the wound tissue of porcine for 2, 3, and 4 weeks postoperatively

To further evaluate the function of the SA/Gel/C scaffold for wound healing and skin reconstruction, the skin tissues were stained by Hematoxylin & Eosin. The results revealed that the degrees of epithelialization, maturation, and tissue regeneration was increased with wound healing after 4 weeks post-operation (Fig. 5a). The process of wound healing includes the formation of granulation tissue on the surface, followed by a thin discontinuous layer of squamous epithelium. Eventually, the entire wound area was covered by nascent tissue. The granulation tissue of the wound had been formed in the scaffold group after 2 weeks post-operation, and the epithelial cells also began to crawl. A thin epithelial layer was formed on the wound surface after 3 weeks post-operation. In addition, the reticulate epithelium on the wound had grown obviously after 4 weeks post-operation. Compared with the scaffold group, the wound healing of the untreated group was delayed. The granulation tissue of the wound had already formed after 2 weeks post-operation. However, the scab had formed on the wound surface in the control group. It showed the sign of wound healing from all sides to the center at 4 weeks. However, the parts of the wound in the middle were incompletely covered with the epidermis. The inconsistent coverage of the thinner epidermis had formed on the untreated group. The noticeable epithelial rete peg protrusions into the dermal area have lacked. Masson’s trichrome staining showed that the collagen fibers were much higher in the scaffold group after 4 weeks post-operation (Fig. 5b). The dermis of wounds in the scaffold group appeared more mature than for the control group. The results further proved that 3D printed SA/Gel/C scaffold was feasible and effective for inhibiting the contracture of skin wounds and the regeneration of the skin.

### Immunofluorescence staining

**Fig. 6:**
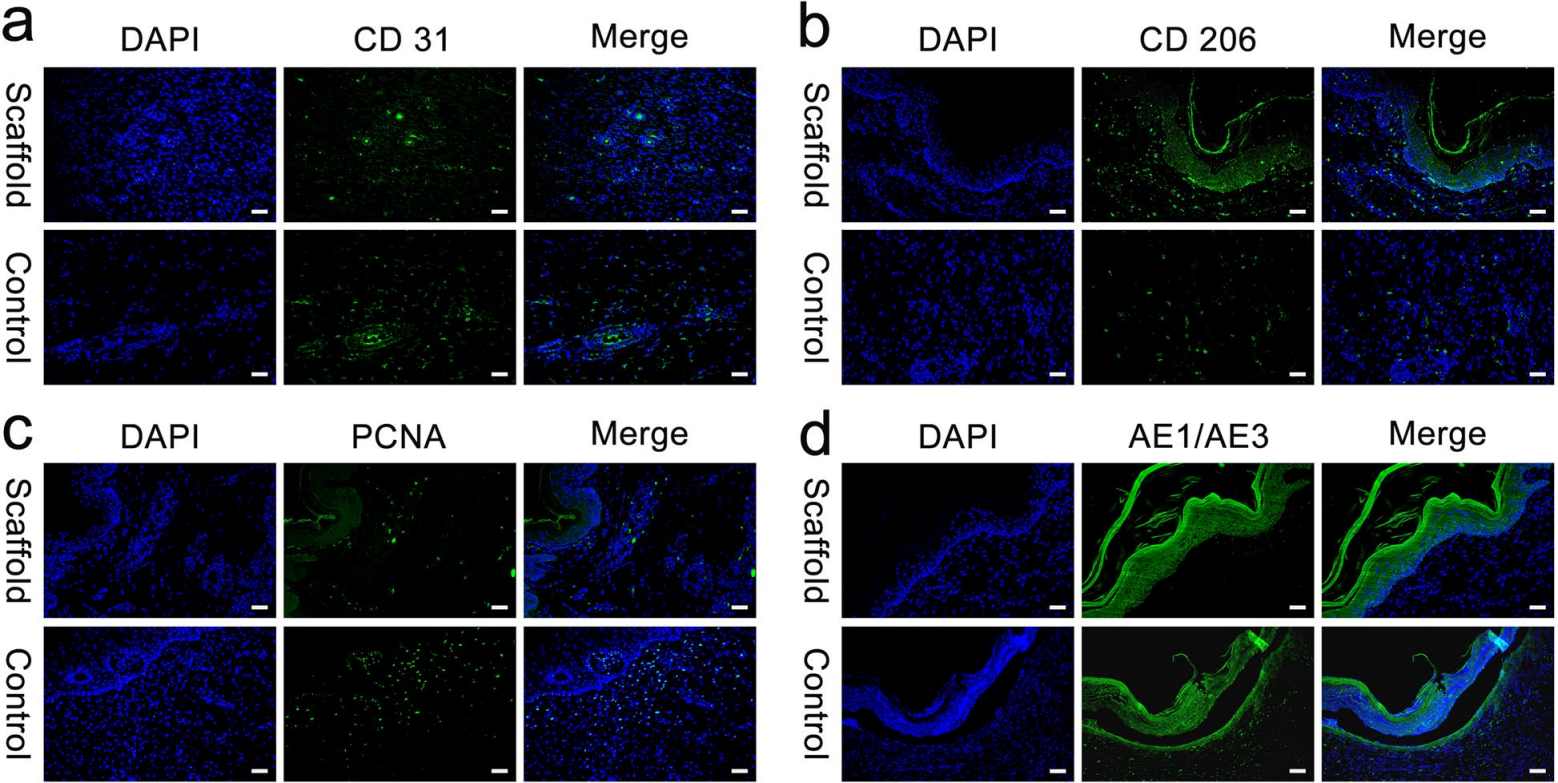
Representative immunofluorescence images of cell nuclei (blue), angio-biomarkers CD31 (green) (**A**), CD206 (green) (**B**), PCNA (green) (**C**), and Cytokeratin AE1/AE3 (green) (**D**) in the control group and the scaffold group, respectively. Scale bars, 50 μm

The effect of the SA/Gel/C scaffold on angiogenesis was investigated (Fig. 6a). The scaffold-treated group showed more CD31-positive cells and more blood vessels than the control group. The CD206 staining for the expression of M2-type microglia/macrophages showed no significant difference between the scaffold repaired skin and the control group (Fig. 6b). To further demonstrate the proliferation of fibroblasts, the expression of PCNA was evaluated by immunofluorescence. The results indicated that the level of PCNA was up-regulated in the control group. However, the level of PCNA was significantly reduced after the treatment of the SA/Gel/C scaffold (Fig. 6c). The results of the scaffold group were a repair without fibrosis and a complete regeneration in the epithelial layer. Compared with the control group, there were many expressions of AE1/AE3 (Fig. 6d).

## Discussion

From the perspective of bio-inks, it is extremely urgent for 3D bioprinting to develop bioinks with high biocompatibility and printability. (Rastin et al., 2020). Therefore, the characterization of bioinks with physical, chemical, and physiological properties and the optimization of formulation parameters are of great significance. Sodium alginate has the advantages of excellent biocompatibility, non-toxicity, and easy crosslinking. There have been some reports that sodium alginate hydrogel maintains the humidity of the physiological environment, which absorbs excess wound fluid. And the disease incidence of wound infection is reduced (Aderibigbe and Buyana, 2018, Lee and Mooney, 2012). Due to its excellent wound healing properties, the wound dressings of sodium alginate hydrogel have been manufactured in many of types. Moreover, the prominent feature of sodium alginate is crosslinked with Ca^2+^ to provide mechanical properties. As a result, the stability and shape fidelity of the scaffold is maintained. However, the biological inertness of sodium alginate also limits the adhesion, extension, and migration of cells (Yao et al., 2019). Despite this, sodium alginate still mimicked the skin matrix components. The active sites for cell adhesion are provided excellently by gelatin and collagen. This study proved that the importance of the optimized hydrogel formulations with stable printability and good mechanical properties for the porous gradient skin scaffold was determined, which promoted re-epithelialization and wound repair.

As the organ with the largest area of the human body, the skin has a complex multilayer structure that is composed of three layers of epidermis, dermis, and subcutaneous tissue from outside to inside. The outermost layer is the epidermis composed of keratinocytes; then the middle layer is the dermis composed of collagen and fibroblasts; finally, the innermost layer is the subcutaneous tissue composed of fat cells and collagen (Vijayavenkataraman et al., 2016). The full-thickness skin tissue engineering scaffold includes both the epidermal layer substitute and the dermis layer substitute. At present, the existing double-layer artificial skin scaffolds on the market are mainly Integra (Dantzer and Braye, 2001, Dantzer et al., 2001) and PELNAC (Suzuki et al., 2000), and their epidermal layers are replaced with a silicone layer. The functions of the silicone layer are protecting the wound surface, preventing infection, retaining water and moisture, and maintaining the moist environment of the wound. However, the degradation of silicone is unfriendly. The degree of inflammatory infections and the suffering for patients with changed dressing once again are increasing.

In this study, the SA/Gel/C scaffold was constructed by the 3D bioprinting extrusion system based on human skin as a model. Four different ratios of SA/Gel/C hydrogels were screened, and the results showed that Gel 1, Gel 2, and Gel 3 were extruded uniformly and stably on this printing platform. In addition, the stability and shape fidelity of the SA/Gel/C scaffold structure was maintained by crosslinking with Ca^2+^. Gelatin is a temperature-sensitive material: the lower the ambient temperature, the more likely it is to form a gel. Due to the high proportion of gelatin in Gel 4, the viscosity increased rapidly under the influence of printing temperature, resulting in uneven extrusion volume. Therefore, the suitable formulation of hydrogel bioinks is indispensable for the shape and structure of the printed scaffold. The printing parameters of printing speed, nozzle height is much easier to adjust. However, it is noted that the SA/Gel/C bioinks are affected greatly by environmental temperature. The printing status of hydrogel is not easy to control. Thus, a well-designed temperature control device (heating tube and cooling platform) installed on the printing platform plays a vital role for some temperature-sensitive materials.

The morphological analysis of the SA/Gel/C scaffold showed that the dense layer surface without macroporous structure protected the wound surface and maintained the moist environment of the wound. On the other side, the epidermis and dermis layer constructed with different proportions of bioink had uniform macroscopic penetrating pores. The printed scaffold with uniform microscopic pores on the surface and inside provided for cell adhesion and growth. Studies reported that the scaffold with a microporous structure built a microenvironment to promote cell migration and the transportation of nutrients such as oxygen, thereby accelerating skin tissue regeneration and tissue structure formation (Griffin et al., 2015). Besides, it was evident that the regeneration capability of the dermis and epidermis was achieved during the repair process. The results showed that the contracture around the wound in the scaffold group was smaller in the control group. And the degrees of epithelialization, maturation, and tissue regeneration was increased with wound healing after 4 weeks post-operation. The collagen fibers, blood vessels were much higher in the scaffold group. The complete regeneration of the epithelial layer has been presented in the scaffold group. Our above experimental results showed that the SA/Gel/C hydrogel scaffold with gradient pore structure has the potential to be completely degradable and can alleviate the pain of the patient’s secondary dressing change. So, it is of great significance for the repair of skin wounds. Furthermore, it is more efficient to use section staining in order to better observe the growth of cells in the scaffold by a confocal microscope. Our future studies will include DAPI staining and cytoskeletal staining of cells within the scaffold to assess cell functionality.

## Conclusions

In this research, the SA / Gel / C scaffold was fabricated by 3D bioprinting technology. The designed gradient pore structure promoted the perfusion of oxygen/nutrition and the excretion of metabolic waste. Therefore, in vivo studies have demonstrated that wound healing was accelerated and the wound contraction was reduced. In addition, large animals were used in the experiments and a series of treatment methods were constructed to mimic the clinical procedures. The preservation and transportation of skin scaffolds should be simplified conditions via twice freeze-drying technologies. The feasibility of scaffolds was evaluated and the advantage of being more convenient for clinical practice.

## Methods

### Materials

Sodium alginate (from brown algae), Gelatin (from porcine skin, Tape A, gel strength ~ 300 g Bloom) and Collagen (from fish skin), Calcium chloride (CaCl_2_) and other reagents were purchased from Sigma Aldrich (St. Louis, MO). Deionized water is made in the laboratory. Phosphate buffered saline (PBS), Fetal bovine serum (FBS), Penicillin-streptomycin solution (PS) and Dulbecco’s modified Eagle’s medium (DMEM) were obtained from Gibco. All other reagents were of chemical grade.

### Preparation of SA/Gel/C hydrogel bioinks

The powder of gelatin (1.8, 2, 2.3, 4 wt.%), SA (2.5, 2, 1.5, 1 wt.%) and collagen (0.5, 1, 0.5, 1 wt.%) were weighed on an electronic analytic balance to prepare SA/Gel/C hydrogels. Then they were mixed with deionized water in a centrifuge tube and dissolved at 37 °C. These bioinks were named as Gel 1(1.8% gelatin, 2.5% SA and 0.5% C), Gel 2 (2% gelatin, 2% SA and 1% C), Gel 3 (2.3% gelatin, 1.5% SA and 0.5% C), Gel 4 (4% gelatin, 1% SA and 1% C), respectively. The sodium alginate and gelatin solution have a certain viscosity, and bubbles will be generated upon shaking. Therefore, the centrifuge tube (bottle mouth facing up) was still placed at 37 °C to allow the liquid stuck on the bottle wall to flow back into the bottle after dissolution and remove the bubbles. The 5 wt.% of CaCl_2_ solution was configured to atomize and crosslink during the printing process. Finally, the scaffold was fully soaked and crosslinked for 30 min at room temperatures after printing.

### 3D printing of SA/Gel/C scaffold

**Fig. 7:**
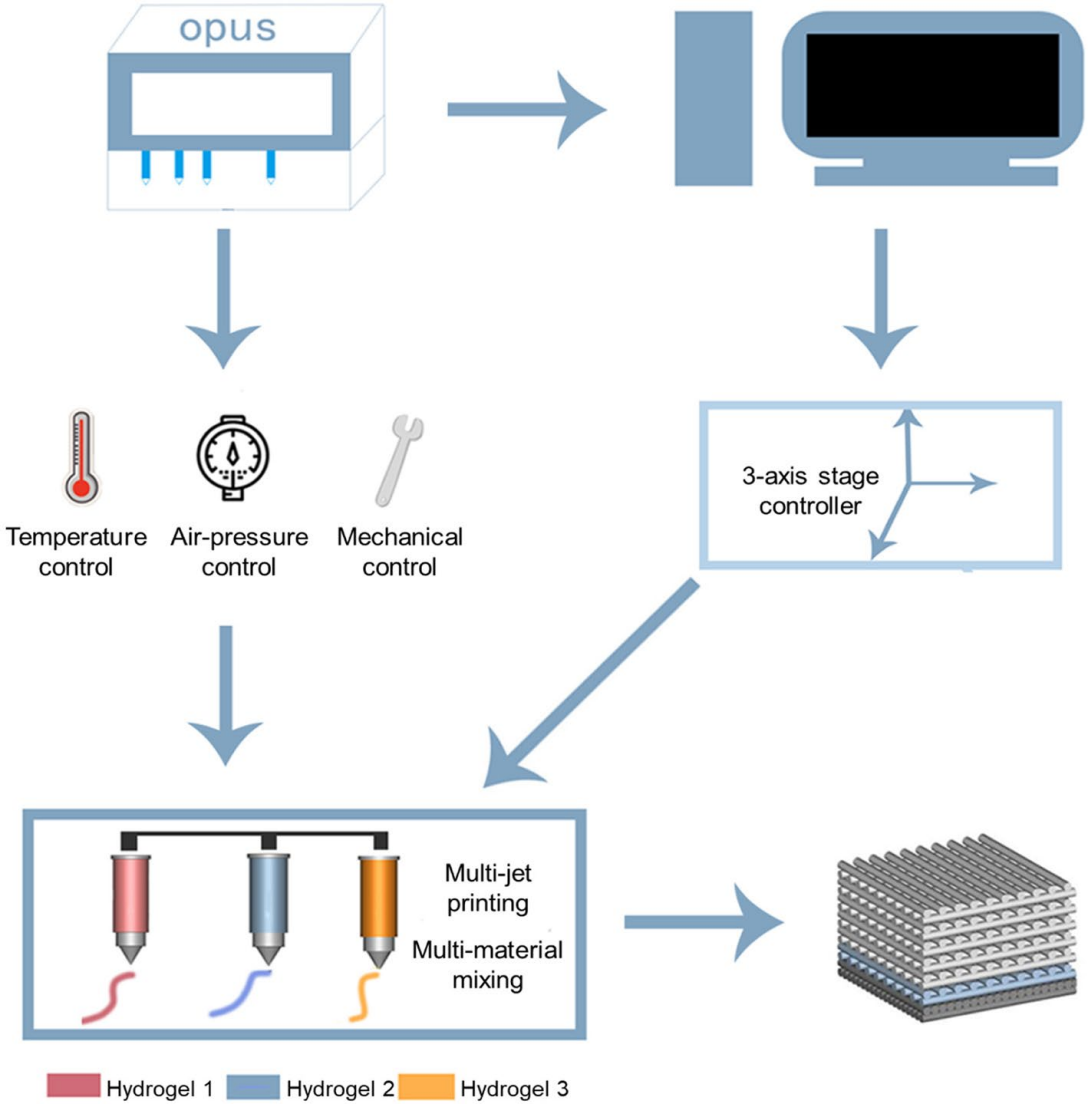
Schematic diagram of the Organ Printing United System (OPUS) and printing process. The foremost part of the system has a computer-aided design system and a three-axis controller, including temperature, air pressure, mechanical control, and a cartridge unit loaded with bioink

As illustrated in Fig. 7, there are three major parts: a computer-aided design system, a three-axis controller (including temperature, air pressure, and mechanical control), and a cartridge unit loaded with biological ink for the self-developed Organ Printing United System (OPUS) printing system. In particular, the integration of printing platforms includes multi-nozzle printing and multi-material mixing, and the hydrogel inks deposited layer by layer were performed on the vessel. The end of each ink cartridge connects to a micro-scale nozzle. The top connects to a pressure controller for squeezing out the bioink and distributing the volume of extrusion. The SA/Gel/C hydrogel bioinks were put into a special syringe at room temperature. When the bubbles disappeared, the equipment was adjusted to the state of printing according to the previously designed structure. The scaffold’s model was a lattice-rod structure with 5 cm × 5 cm × 2.1 mm, which was converted into a G-Code file using Slic3r with a layer height of 0.15 mm (14 layers). And with an inner diameter of 500 μm of the printing nozzle was selected. The SA/Gel/C scaffolds were printed in a layer-by-layer deposition fashion on the bioprinter platform. The printability temperature window of the bioink was at 15–20 °C. The temperature of the bioink was about 19 °C after printing.

### Scanning electron microscopy of cell and scaffold morphology

The morphology of the scaffold without cells was investigated using desktop scanning electron microscopy (SEM) (Phenom ProX, 5 kV electron beam; the Netherlands). Briefly, the scaffolds were frozen at 4 °C and − 80 °C for 2 h, respectively, and then lyophilized overnight for the first time in a vacuum freeze-dryer (SCIENTZ-10 N, Ningbo, China). Next, the freeze-dried samples were cross-linked with 5% CaCl_2_ for 30 min and washed thoroughly with sterile water. The hydrogels were placed at − 80 °C for 2 h and freeze-dried and stored in a vacuum container. They were immersed in liquid nitrogen to quench and coated with a 10 nm gold film before the SEM measurement.

The morphology of the scaffold with cells was observed by SEM. Fibroblasts were mixed with the hydrogel solution at a density of 2 × 10^6^ cells/ml and incubated at 5% CO_2_ and 37 °C for 1 day. The samples were immobilized with a 2.5% glutaraldehyde solution at 4 °C for 30 min. Then they were placed at − 80 °C for 2 h and freeze-dried and stored in a vacuum container before being coated with a 10 nm gold film before the SEM measurement.

### Live/dead cell staining

Human fibroblasts were obtained from the human foreskin dermal tissue from the National Collection of Authenticated Cell Culture. The cells were cultured in a T-175 flask (Corning, USA) with high-glucose Dulbecco’s modified Eagle’s medium (DMEM, Hyclone, UT) supplemented with 10% fetal bovine serum (FBS, Gibco, USA) and 1% penicillin-streptomycin in an incubator (37 °C, 5% CO_2_). The complete medium was updated every two days. The passage 5 to 8 cells were used for experiments. The hydrogel was mixed with fibroblasts (cell density: 2 × 10^6^ cells/ml). Cell viability was identified by 2 μM Calcein AM (live cell stain, 490 nm) and 4 μM propidium iodides (dead cell stain, 535 nm) (PI) (Dojindo, Tokyo, Japan) at 0, 3, and 6 days, respectively. According to the manufacturer’s instructions, the living and dead cells were labeled in green and red, respectively. Samples were washed three times PBS and stained with Calcein AM and PI for 30 min in the dark. The images were acquired through a fluorescence microscope with an imaging system (IX73, Olympus, Japan). The number of green and red stain cells was counted using Image J visualization software (National Institutes of Health, Bethesda, MD, USA).

### MTT assay

Cell proliferation was tested by using the MTT assay at days 0, 3, and 6. Fibroblasts (cell density: 5 × 10^5^ cells/ml) on scaffold were incubated with 5 mg/mL MTT (Sigma-Aldrich, USA) for 4 h. Then, the medium was transferred into wells of a 96-well plate. The absorbance of each well was measured at 570 nm using a microplate reader (Varioskan Flash, Thermo Fisher Scientific, United States).

### Porcine full-thickness wound model

The healing of 3D bioprinted scaffolds in vivo was evaluated by creating full-thickness skin wounds on the back surface of pigs. The weight of pigs was 20–35 kg. They were anesthetized using intravenous injection of 4% pentobarbital sodium at 0.2 mL kg^− 1^, then fixed on the operating table in a prone position. Next, routine skin preparation and disinfection were performed. The surgical areas were shaved using an electric razor and tattooed by a tattoo pen to mark the area of the excisional wound. The wounds were washed with 75% alcohol and iodine. Six skin defects were created symmetrically deep into the panniculus carnosus layer with a length of 4 cm and a width of 4 cm in size on the pig’s back. After thorough debridement to stop bleeding, the wound was repeatedly washed with gentamicin and covered with sterile saline gauze. In the scaffold group, the 3D bioprinted scaffolds pre-soaked in 0.9% sodium chloride injection for 10 min were implanted into the wound (*n* = 3). Scaffolds were sutured and fixed on the edge of the skin, covered with petrolatum gauze, and then wrapped sterile gauze on the outer layer. The control group was directly covered with petrolatum gauze and sterile gauze, then bandaged with an elastic bandage (*n* = 3). Tissue blocks with a diameter of 4 mm were taken using a biopsy punch from the wound at different time points postoperatively. All experiments were performed following Animal Care and Use Committee guidelines and regulations.

### Histology analysis

To evaluate the epithelium and collagen distribution of the skin tissue, histological sections were prepared and stained with hematoxylin and eosin (H&E) and Masson’s trichrome staining. The full-thickness punch biopsies (4–6 mm) were taken from every treatment wound in the center and the edge parts at weeks 2, 3, and 4. The specimens were immediately fixed in 4% paraformaldehyde solution for 4 h at room temperature. The samples were dehydrated and cleared through graded alcohols and finally embedded in the paraffin blocks. Afterward, the specimens were imaged and observed by an optical microscope.

### Immunofluorescence staining

The sections were processed for immunofluorescence for CD31 (1:100, Proteintech Group, Inc), CD206 Monoclonal Antibody (1500, Proteintech Group, Inc), AE1/AE3 (Santa Cruz Biotechnology, Inc.), Proliferating Cell Nuclear Antigen (PCNA, 1:500, Abcam plc) antibody. Briefly, the sections were incubated with primary antibodies at 4 °C overnight. After washing with phosphate-buffered saline (PBS), a secondary antibody (FITC) was used at 37 °C for 45 min in the dark. The sections were counterstained with DAPI at room temperature for 10 min in the dark. The sections were observed and collected under an inverted fluorescence microscope imaging system.

## Data Availability

All data supporting the findings of this study are available within the article and its supplementary information files or from the corresponding upon reasonable request.

## Abbreviations

SA: sodium alginate
FF: freeform fabrication
OPUS: Organ Printing United System
HE: Hematoxylin & Eosin
PBS: Phosphate buffered saline
FBS: Fetal bovine serum
PS: Penicillin-streptomycin
DMEM: Dulbecco’s modified Eagle’s medium
SEM: scanning electron microscopy
